# Association of eNOS T786C genetic polymorphism with the risk of aneurysmal subarachnoid haemorrhage

**DOI:** 10.1515/tnsci-2025-0368

**Published:** 2025-04-16

**Authors:** Josip Ljevak, Kristina Gotovac Jerčić, Antonela Blažeković, David Ozretić, Ivan Perić, Nikola Blažević, Fran Borovečki, Zdravka Poljaković Skurić

**Affiliations:** Department of Neurology, University Hospital Center Zagreb, 10 000, Zagreb, Croatia; Department for Personalized Medicine, University Hospital Center Zagreb, 10 000, Zagreb, Croatia; Department for Anatomy and Clinical Anatomy, University of Zagreb School of Medicine, 10 000, Zagreb, Croatia; Department of Neuroradiology, University Hospital Center Zagreb, University of Zagreb School of Medicine, 10 000, Zagreb, Croatia; Department for Functional Genomics, Center for Translational and Clinical Research, University of Zagreb School of Medicine, University Hospital Center Zagreb, 10 000, Zagreb, Croatia; Department of Neurology, University of Zagreb School of Medicine, 10 000, Zagreb, Croatia

**Keywords:** aneurysm, subarachnoid haemorrhage, eNOS, polymorphism

## Abstract

**Background:**

Unruptured intracranial aneurysms (IAs) are increasingly detected due to advancements in neuroimaging. Despite improvements in treatment, aneurysmal subarachnoid haemorrhage (aSAH) is associated with high mortality and morbidity. Treatment decisions for IA are complex and individualized, considering aneurysm and patient-related risk factors. Genetic factors, particularly endothelial nitric oxide synthase (eNOS) polymorphisms, have been implicated in IA formation and rupture risk.

**Methods:**

This study investigated the association between three eNOS polymorphisms (27-bp-VNTR, T786C, and G894T) and aSAH in a cohort of 275 patients with unruptured IA or aSAH. Patients were followed for at least 8 years with clinical and imaging assessments. Genotyping of selected polymorphisms was performed, and statistical analyses were conducted to identify interactions between genetic polymorphisms and established risk factors.

**Results:**

A significant difference in the frequencies of genotypes and allele carriers of the T786C polymorphism was observed between patients with unruptured IA and those with aSAH, with an increased proportion of CC homozygotes in the aSAH group. The risk of rupture was higher in patients with the CC genotype. Multilobular aneurysms and those located in the posterior circulation had a higher incidence of rupture. Associations between arterial hypertension and certain genotypes were also found. However, no significant interaction was observed between the polymorphisms and established risk factors in relation to aneurysm rupture.

**Conclusion:**

Our data showed a significant and independent correlation between eNOS genetic polymorphism T786C and aSAH.

## 1 Introduction

Technical advancement and availability of neuroimaging, primarily magnetic resonance imaging, have made the detection of unruptured intracranial aneurysms (IA) easier and more common in routine clinical practice. Despite improvements in both endovascular and neurosurgical treatment, as well as in neurocritical care, aneurysmal subarachnoid haemorrhage (aSAH), as the worst outcome of IA, is associated with high mortality (20–30%) and morbidity [1,2]. Treatment of unruptured IA, either neurosurgical or endovascular, carries an inherent risk of complications, with morbidity and mortality rates as high as 5% [3]. Consequently, clinical decisions on preventive occlusion of unruptured IA need to be nuanced and highly individualized.

Clinical scores [3] and current society guidelines [4] recommend considering several types of risk factors for aSAH, which can be roughly divided into aneurysm-related and patient-related factors. The established risk factors used in the study population were as follows:

- female sex;
- aneurysm growth is defined as an increase in aneurysm size >1 mm in any diameter;
- arterial hypertension (AH) defined as one of the following: (a) previously established diagnosis of AH, (b) initiation of antihypertensive therapy during the follow-up period, and/or (c) repeated measurements of blood pressure (BP) 140/90 mmHg or higher;
- any smoking (current or previous);
- aneurysm size >4 mm in largest diameter;
- multilobular aneurysm morphology;
- IA in posterior cerebral circulation (vertebral, basilar, and posterior cerebral artery).

Hypertension, sex, and smoking were considered patient-related risk factors, while we considered IA growth, location, morphology, and size as aneurysm-related risk factors.

Population-based differences in aSAH incidence without a difference in IA prevalence, increased risk of IA in certain inherited conditions (e.g. autosomal dominant polycystic kidney disease), and higher risk of a positive family history of aSAH suggests a genetic component in aSAH development [5]. This genetic association has been evaluated in numerous published trials, but mostly on IA formation [6]. The proportion of these studies pertained to endothelial nitric oxide genetic (eNOS) polymorphisms, with conflicting and seemingly population-dependent results [7].

**Figure 1:**
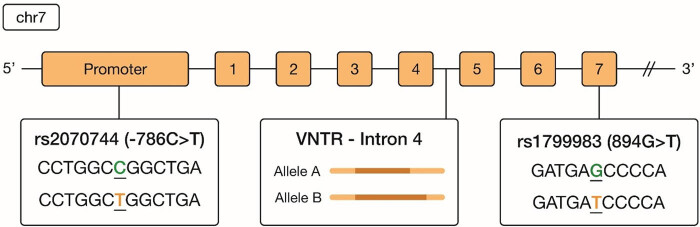
Scheme of the gene encoding endothelial nitric oxide synthase (eNOS). Exons are described by numbers. Location of three eNOS polymorphisms added in squares.

The *eNOS* gene, also known as *NOS3,* is located on chromosome 7 in humans. This gene encodes for the eNOS enzyme, which is crucial for the production of nitric oxide (NO), a molecule that plays a significant role in vascular homeostasis [8]. The gene consists of 26 exons and spans approximately 21 kilobases (kb) in length. There are several functional polymorphisms in different regions of *eNOS*, and various studies have explored the relationship between these polymorphisms and susceptibility to IA. The three most clinically relevant IA-associated polymorphisms in *eNOS* that have been reported are the 27-bp-VNTR, T786C, and G894T [9]. The 27-bp VNTR polymorphism occurs in intron 4 of the *eNOS* gene and is characterized by a variable repeat sequence of 27 base pairs (Figure 1). The 27-bp-VNTR polymorphism affects the transcriptional regulation and functioning of the enzyme. Additionally, variations in plasma NO levels linked to the 27-bp VNTR may lead to endothelial dysfunction, further predisposing individuals to vascular pathologies such as IA [10]. The T786C polymorphism, located in the *eNOS* gene promoter region, results in a thymine (T) to cytosine (C) nucleotide change and has been frequently associated with reduced eNOS activity and lower NO production. This polymorphism results in decreased promoter activity, which directly lowers NO synthesis. Khurana et al. connected the T-786C mutation with an increased risk of vasospasm following aSAH [10]. The G894T polymorphism, found in exon 7, results in a substitution of glutamate for aspartate at position 298 (Glu298Asp). Reduced NO production associated with the G894T variant aligns with findings that suggest this polymorphism may heighten the risk of intra- and post-operative complications related to IAs [11]. Carriers of the T allele show altered endothelial function, which could be critical during IA development, where endothelial dysfunction is a known contributing factor [12].

Current guidelines recognize the difficulties in clinical practice regarding the optimal selection of patients for prophylactic treatment, often resulting in low-quality evidence and weak recommendations based on the current literature while encouraging additional research [4]. Additionally, genetic susceptibility of aSAH is widely recognized but incompletely defined outside of several specific monogenic disorders. Finally, studies of the correlation of eNOS polymorphisms and IA formation or rupture seem to yield conflicting results.

We conducted an association study between three eNOS genetic polymorphisms (27-bp-VNTR, T786C, and G894T) and aSAH in our hospital. Statistical analysis was performed to identify possible interactions between the genetic polymorphisms and established risk factors.

## 2 Methods

### 2.1 Study population and blood sampling

Subjects involved in the study were patients treated in the Intensive Care Unit of the Department of Neurology (Clinical Hospital Center Zagreb) – a total of 275 patients. The median age of our patients was 53 years. The study was conducted in accordance with the Declaration of Helsinki. After signing the informed consent to participate in the study, the subjects were divided into two groups: patients with unruptured IA and patients with aSAH. We included patients with IA diagnosis confirmed by digital subtraction angiography (DSA); the presence of aSAH was diagnosed by non-invasive imaging or lumbar puncture with cerebrospinal fluid analysis. Patients were included after signing informed consent and all were older than 18 years. The main exclusion criteria were negative DSA (no signs of IA, regardless of the presence of subarachnoid bleed) and lack of informed consent. Aneurysm growth was defined as an increase >1 mm of aneurysm diameter in any direction on repeat imaging. For our study, we defined hypertension as either previously diagnosed AH, the need for starting antihypertensive therapy, and/or repeat BP measurements of 140/90 mmHg or higher. Smoking was defined as any sustained smoking previously and/or current smoking. Based on clinical experience of treating aSAH, we used an IA size cut-off of >4 mm for risk stratification. The morphology of IA was described by an experienced interventional neuroradiologist based on DSA, with IA showing signs of more than one lobule considered morphologically higher risk for rupture. Posterior circulation location was defined as IA originating on vertebral, basilar, posterior cerebral, and/or posterior communicating arteries or branches of these arteries. Clinical monitoring, as well as the decision on the possible treatment of verified aneurysms, was made in accordance with the current guidelines of international neurological or interdisciplinary societies [4] and in accordance with the usual clinical practice of the neurovascular indication team from the Clinical Hospital Center Zagreb. For each subject, 5 ml of venous blood was collected in a test tube with EDTA (ethylenediaminetetraacetic acid) for DNA isolation.

### 2.2 DNA analysis and genotyping

Genomic DNA was isolated using a commercial reagent kit (Qiagen DNAeasy) according to the manufacturer’s instructions. The concentration and quality of isolated DNA were determined using a spectrophotometer NanoDrop 2000 (Thermo Fisher Scientific). Nucleotide changes were determined by PCR-restriction fragment length polymorphism analysis, using isolated genomic DNA as a template. Polymorphisms (eNOS 27-bp-VNTR, eNOS T786C, and eNOS G894T) were detected using a previously described protocol [9]. The primers used for the PCRs are listed in Table 1. PCR products were separated by electrophoresis on a 3% agarose gel in 1XTAE buffer (Tris-acetate-EDTA: 0.045M Tris-acetate, 0.001 M EDTA, pH 8.0) stained with ethidium bromide and visualized using a UV-transilluminator.

**Table 1:** Primer sequences for each of the three *eNOS* polymorphisms (F – forward primer, R – reverse primer)

Gene polymorphism	Primer sequence
*eNOS 4b/4a*	F: 5′-AGGCCCTATGGTAGTGCCTTT-3′
	R: 5′-TCTCTTTAGTGCTGTGGTCAC-3′
*eNOS G894T*	F: 5′-CATGAGGCTCAGCCCCAGAAC-3′
	R: 5′-AGTCAATCCCTTTGGTGCTCAC-3′
*eNOS T786C*	F: 5′-ATGCTCCCACCAGGGCATCA-3′
	R: 5′-GTCCTTGAATCTGACATTAGGG-3′

### 2.3 Statistical analysis

Statistical analyses were performed using SPSS software (IBM, USA). Since the distribution of subjects’ age was not normal (*p* < 0.05, Kolmogorov–Smirnov test), a non-parametric Mann–Whitney test was used for further analysis of that variable, and the values were presented as the median and minimal and maximal values. The *χ* ^2^ test was used to compare the distribution of the genotypes, haplotypes, and other categorical variables. The linkage disequilibrium of *eNOS* alleles for polymorphisms 27-bp-VNTR, T786C, and G894T was tested using Haploview 4.2. software (Broad Institute of Harvard and MIT, USA) [13]. The same program was used to check the deviation of the genotypes’ distribution from the Hardy–Weinberg equilibrium. The haplotype pairs for each subject were estimated using gPLINK 2.050 software [14]. To determine the joint influence of polymorphisms/haplotypes and selected risk factors on the incidence of aSAH, logistic regression was used, with aSAH as the dependent variable and the combination of polymorphism/haplotypes and each individual factor as independent variables. The α significance level was set at 0.05, and all tests were two-tailed.


**Ethics approval:** The research related to human use has complied with all the relevant national regulations, institutional policies and in accordance with the tenets of the Helsinki Declaration and has been approved by the author’s institutional review board or equivalent committee. The research was conducted with written approval obtained from the local ethics committee (University Hospital Center Zagreb).
**Informed consent:** Informed consent has been obtained from all individuals included in this study.

## 3 Results

### 3.1 General features

A total of 275 patients with IA were analysed. The age of the patients ranged from 19 to 83 years, with a median age of 53 years. The clinical characteristics of the patients included in the study are summarized in Table 2. In the group of patients with aSAH median age was 52 years, the proportion of ruptured IA with a diameter ≤4 mm was 46.5, and 64.1% were multilobular.

**Table 2:** Demographic characteristics, traditional risk factors, and aneurysm details

Risk factor		Number of subjects (percentage)
aSAH	No	154 (56.0%)
	Yes	121 (44.0%)
Aneurysm growth	No	262 (95.3%)
	Yes	13 (4.7%)
Hypertension	No	123 (44.7%)
	Yes	152 (55.3%)
Smoking	No	164 (59.6%)
	Yes	111 (40.4%)
Aneurysm size >4 mm	No	86 (31.3%)
	Yes	189 (68.7%)
Multilobular aneurysm	No	236 (85.8%)
	Yes	39 (14.2%)
Posterior location	No	219 (79.6%)
	Yes	56 (20.4%)
Gender	Male	85 (30.9%)
	Female	190 (69.1%)

aSAH, aneurysmal subarachnoid haemorrhage.

A total number of patients with IA-275. The age of the patients ranged from 19 to 83 years (median age: 53).

In the examined sample, there were no significant differences between men and women in the occurrence of aSAH (*χ* ^2^ = 0.14; *p* = 0.713) or the distribution of genotypes according to polymorphisms 27-bp-VNTR (*χ* ^2^ = 1.25; *p* = 0.535), T786C (*χ* ^2^ = 1.29; *p* = 0.524), and G894T (*χ* ^2^ = 0.87; *p* = 0.648). Therefore, further analyses were done together for men and women.

The Mann–Whitney test showed that patients with AH were significantly older (*U* = 12914.50; *p* < 0.001) than patients without AH. In addition, in the studied sample, women were significantly (*U* = 9674.50; *p* = 0.009) older than men. No difference in age was observed when patients were divided according to the other tested parameters: aSAH (*U* = 8845.00; *p* = 0.471), aneurysm growth (*U* = 1940.50; *p* = 0.396), smoking (*U* = 8539.00; *p* = 0.384), aneurysm size (*U* = 8954.50; *p* = 0.176), multilobular aneurysm (*U* = 3974.00; *p* = 0.172), and posterior location (*U* = 6852.00; *p* = 0.175).

Two aneurysm-related factors were found to be significantly different between the groups. Multilobular IA (*χ* ^2^ = 7.45; *p* = 0.006, *R* = 1.89) and IA located in posterior circulation (*χ* ^2^ = 4.93; *p* = 0.026, *R* = 1.48) were more common in the aSAH group. Other examined factors had no influence on the incidence of aneurysm rupture: hypertension (*χ* ^2^ = 0.08; *p* = 0.784), smoking (*χ* ^2^ = 0.00; *p* = 0.968), and aneurysm size (*χ* ^2^ = 0.32; *p* = 0.571). To further examine a possible combined effect of smoking and hypertension, logistic regression analysis (*χ* ^2^ = 0.043; *p* = 0.835) was performed, indicating the lack of this combined effect on the incidence of rupture (*B* = 0.06; *p* = 0.835).

### 3.2 Polymorphisms 27-bp-VNTR, G-894T, and T-786C of the *eNOS* gene in patients with IA

The genotypic frequencies of the polymorphisms included in this study and the results of the association analysis between patients with unruptured IA and aSAH are summarized in Table 3. Three different eNOS polymorphisms were analysed from each blood sample. The distribution of genotypes and allele carriers for 27-bp-VNTR and G894T polymorphisms did not differ significantly between the groups. There was a significant difference (*χ* ^2^ = 8.04; *p* = 0.018) in the genotype distribution of the T786C polymorphism between patients with unruptured IA and aSAH. In addition, there was a significant difference (*χ* ^2^ = 6.00; *p* = 0.014) in the distribution of T allele carriers and CC homozygotes regarding the T786C polymorphism between patients with unruptured IA and aSAH. In addition, when dividing patients into CC homozygotes and carriers of the T allele, the odds ratio (OR) (95% CI) was 2.664 (1.189–5.968), indicating that the risk of aSAH was significantly higher in the group of patients with the homozygous CC genotype.

**Table 3:** Genotype distribution and allele frequencies of *eNOS* 27-bp-VNTR, G894T, and T786C polymorphisms between patients with unruptured IA (No-aSAH) and aSAH

Polymorphism	Genotype (*N*; %)	Number of subjects *N* (%)	
		No-aSAH	aSAH
27VNTR (*N* = 275)	4a4a (5; 1.8)	2 (1.3)	3 (2.5)
	4a4b (76; 27.6)	38 (24.7)	38 (31.4)
	4b4b (194; 70.6)	114 (74.0)	80 (66.1)
	No-SAH vs SAH	*χ* ^2^ = 2.23; *p* = 0.328	
	4b4b homozygotes (194; 70.6)	114 (74.0)	80 (66.1)
	a carriers (4a4a + 4a4b) (81; 29.4)	40 (26.0)	41 (33.9)
	No-SAH vs SAH	*χ* ^2^ = 2.04; *p* = 0.153	
	4a4a homozygotes (5; 1.8)	2 (1.3)	3 (2.5)
	b carriers (4b4b + 4a4b) (270; 98.2)	152 (98.7)	118 (97.5)
	No-SAH vs SAH	*χ* ^2^ = 0.53; *p* = 0.467	
G894T (*N* = 264)	GG (80; 30.3)	49 (33.8)	31 (26.1)
	GT (136; 51.5)	71 (49.0)	65 (54.6)
	TT (48; 18.2)	25 (17.2)	23 (19.3)
	No-SAH vs SAH	*χ* ^2^ = 1.86; *p* = 0.395	
	TT homozygotes (48; 18.2)	25 (17.2)	23 (19.3)
	G carriers (GG + GT) (216; 81.8)	120 (82.8)	96 (80.7)
	No-SAH vs SAH	*χ* ^2^ = 0.19; *p* = 0.662	
	GG homozygotes (80; 30.3)	49 (33.8)	31 (26.1)
	T carriers (TT + GT) (184; 69.7)	96 (66.2)	88 (73.9)
	No-SAH vs SAH	*χ* ^2^ = 1.86; *p* = 0.173	
T786C (*N* = 274)	CC (29; 10.6)	10 (6.5)	19 (15.7)
	CT (131; 47.8)	82 (53.6)	49 (40.5)
	TT (114; 41.6)	61 (39.9)	53 (43.8)
	No-SAH vs SAH	*χ* ^2^ = 8.04; ***p*** **= 0.018**	
	TT homozygotes (114; 41.6)	61 (39.9)	53 (43.8)
	C carriers (CC + CT) (160; 58.4)	92 (60.1)	68 (56.2)
	No-SAH vs SAH	*χ* ^2^ = 0.43; *p* = 0.512	
	CC homozygotes (29; 10.6)	10 (6.5)	19 (15.7)
	T carriers (CT + TT) (245; 89.4)	143 (93.5)	102 (84.3)
	No-SAH vs SAH	*χ* ^2^ = 6.00; ***p*** **= 0.014**	

Genotype frequencies are shown as numbers (%). Significant results are marked in bold. *N* = number of patients, aSAH, aneurysmal subarachnoid haemorrhage.

There was no statistical significance in the genotype distribution and allelic frequencies for the tested polymorphisms and development of multilobular aneurysms (Table 4).

**Table 4:** Genotype distribution and allele frequencies of eNOS 27-bp-VNTR, G894T, and T786C polymorphisms between patients according to IA type (saccular or multilobular)

Polymorphism	Genotype (*N*; %)	Number of subjects *N* (%)	
		Saccular IA	Multilobular IA
27VNTR (*N* = 275)	4a4a (5; 1.8)	4 (1.7%)	1 (2.6%)*
	4a4b (76; 27.6)	62 (26.3%)	14 (35.9%)
	4b4b (194; 70.6)	170 (72.0%)	24 (61.5%)
	
	4b4b homozygotes (194; 70.6)	170 (72.0%)	24 (61.5%)
	a carriers (4a4a + 4a4b) (81; 29.4)	66 (28.0%)	15 (38.5%)
		*χ* ^2^ = 1.77; *p* = 0.183	
	4a4a homozygotes (5; 1.8)	4 (1.7%)	1 (2.6%)*
	b carriers (4b4b + 4a4b) (270; 98.2)	232 (98.3%)	38 (97.4%)
	
G894T (*N* = 264)	GG (80; 30.3)	69 (30.7%)	11 (28.2%)
	GT (136; 51.5)	115 (51.1%)	21 (53.8%)
	TT (48; 18.2)	41 (18.2%)	7 (17.9%)
		*χ* ^2^ = 1.12; *p* = 0.944	
	TT homozygotes (48; 18.2)	41 (18.2%)	7 (17.9%)
	G carriers (GG + GT) (216; 81.8)	184 (81.8%)	32 (82.1%)
		*χ* ^2^ = 0,00; *p* = 0.967	
	GG homozygotes (80; 30.3)	69 (30.7%)	11 (28.2%)
	T carriers (TT + GT) (184; 69.7)	156 (69.3%)	28 (71.8%)
		*χ* ^2^ = 0.10; *p* = 0.757	
T786C (*N* = 274)	CC (29; 10.6)	23 (9.8%)	6 (15.4%)
	CT (131; 47.8)	112 (47.7%)	19 (48.7%)
	TT (114; 41.6)	100 (42.6%)	14 (35.9%)
		*χ* ^2^ = 1.35; *p* = 0.508	
	TT homozygotes (114; 41.6)	100 (42.6%)	14 (35.9%)
	C carriers (CC + CT) (160; 58.4)	135 (57.4%)	25 (64.1%)
		*χ* ^2^ = 0,61; *p* = 0,435	
	CC homozygotes (29; 10.6)	23 (9.8%)	6 (15.4%)
	T carriers (CT + TT) (245; 89.4)	212 (90.2%)	33 (84.6%)
		*χ* ^2^ = 1.11; *p* = 0.293	

Genotype frequencies are shown as numbers (%). *N* = number of patients. *Statistical analysis was not performed, as the patient number in the group was too low.

Our results indicate the association between the presence of AH and certain genotypes according to investigated polymorphisms. Although 27-bp-VNTR polymorphism was found to be associated with hypertension (*χ* ^2^ = 7.36; *p* = 0.025), the results are debatable since there were no A homozygotes with hypertension. For the G894T polymorphism, there was a significant difference in the distribution of T and G homozygous carriers between patients with and without AH (*χ* ^2^ = 4.37; *p* = 0.037). The difference was mostly due to the reduced proportion of G homozygotes in the group of patients with normal arterial pressure (*R* = −1.30), which is increased (*R* = 1.17) in the group of subjects with hypertension. In the case of the T786C polymorphism, the significant differences (*χ* ^2^ = 4.04; *p* = 0.044) were mostly due to the increased proportion of C homozygotes (*R* = 1.42) in patients with normal arterial pressure (Table 5).

**Table 5:** Genotype distribution and allele frequencies of eNOS 27-bp-VNTR, G894T, and T786C polymorphisms between patients according to BP

Polymorphism	Genotype (*N*; %)	Number of subjects *N* (%)	
		Normal BP	AH
27VNTR (*N* = 275)	4a4a (5; 1.8)	5 (4.1)	0 (0.0)
	4a4b (76; 27.6)	37 (30.1)	39 (25.7)
	4b4b (194; 70.6)	81 (65.9)	113 (74.3)
		*χ* ^2^ = 7.36; ***p*** **= 0.025**	
	4b4b homozygotes (194; 70.6)	81 (65.9)	113 (74.3)
	a carriers (4a4a + 4a4b) (81; 29.4)	42 (34.1)	39 (25.7)
		*χ* ^2^ = 2.36; *p* = 0.125	
	4a4a homozygotes (5; 1.8)	5 (4.1)	0 (0.0)
	b carriers (4b4b + 4a4b) (270; 98.2)	118 (95.9)	152 (100.0)
		*χ* ^2^ = 6.29; ***p*** **= 0.012**	
G894T (*N* = 264)	GG (80; 30.3)	28 (23.7)	52 (35.6)
	GT (136; 51.5)	63 (53.4)	73 (50.0)
	TT (48; 18.2)	27 (22.9)	21 (14.4)
		*χ* ^2^ = 5.78; ***p*** **= 0.056**	
	TT homozygotes (48; 18.2)	27 (22.9)	21 (14.4)
	G carriers (GG + GT) (216; 81.8)	91 (77.1)	125 (85.6)
		*χ* ^2^ = 3.17; *p* = 0.075	
	GG homozygotes (80; 30.3)	28 (23.7)	52 (35.6)
	T carriers (TT + GT) (184; 69.7)	90 (76.3)	94 (64.4)
		*χ* ^2^ = 4.37; ***p*** **= 0.037**	
T786C (*N* = 274)	CC (29; 10.6)	18 (14.8)	11 (7.2)
	CT (131; 47.8)	48 (39.3)	83 (54.6)
	TT (114; 41.6)	56 (45.9)	58 (38.2)
		*χ*2 = 7.89; ***p*** **= 0.019**	
	TT homozygotes (114; 41.6)	56 (45.9)	58 (38.2)
	C carriers (CC + CT) (160; 58.4)	66 (54.1)	94 (61.8)
		*χ* ^2^ = 1.67; *p* = 0.196	
	CC homozygotes (29; 10.6)	18 (14.8)	11 (7.2)
	T carriers (CT + TT) (245; 89.4)	104 (85.2)	141 (92.8)
		*χ* ^2^ = 4.04; ***p*** **= 0.044**	

Genotype frequencies are shown as numbers (%). Significant results are marked in bold. *N* = number of patients, BP, blood pressure, AH, arterial hypertension.

We examined polymorphisms (27-bp-VNTR, G894T) and risk factors that did not affect the incidence of rupture (smoking, hypertension, gender, and aneurysm size) to determine whether any of their combinations contributed to a higher frequency of rupture. Logistic regression was performed with rupture as the dependent variable and the combination of polymorphism and each factor as independent variables. The results indicated the lack of a significant interaction model of the 27-bp-VNTR and the G894T polymorphisms with selected risk factors (Table 6). No combination of genotypes of tested polymorphisms and selected risk factors had a significant effect on the occurrence of aneurysm rupture.

**Table 6:** Examination of the combined effect of polymorphisms (27-bp-VNTR, G894T) and risk factors that did not affect the incidence of rupture

Risk factor	*eNOS* polymorphism			
	27-bp-VNTR		G894T	
Hypertension	*B* = 0.00	*p* = 0.978	*B* = 0.03	*p* = 0.828
Smoking	*B* = 0.01	*p* = 0.898	*B* = 0.01	*p* = 0.918
Gender	*B* = 0.04	*p* = 0.773	*B* = 0.04	*p* = 0.741
Aneurysm size	*B* = −0.09	*p* = 0.373	*B* = 0.02	*p* = 0.872

B, Logistic regression coefficient.

**Figure 2:**
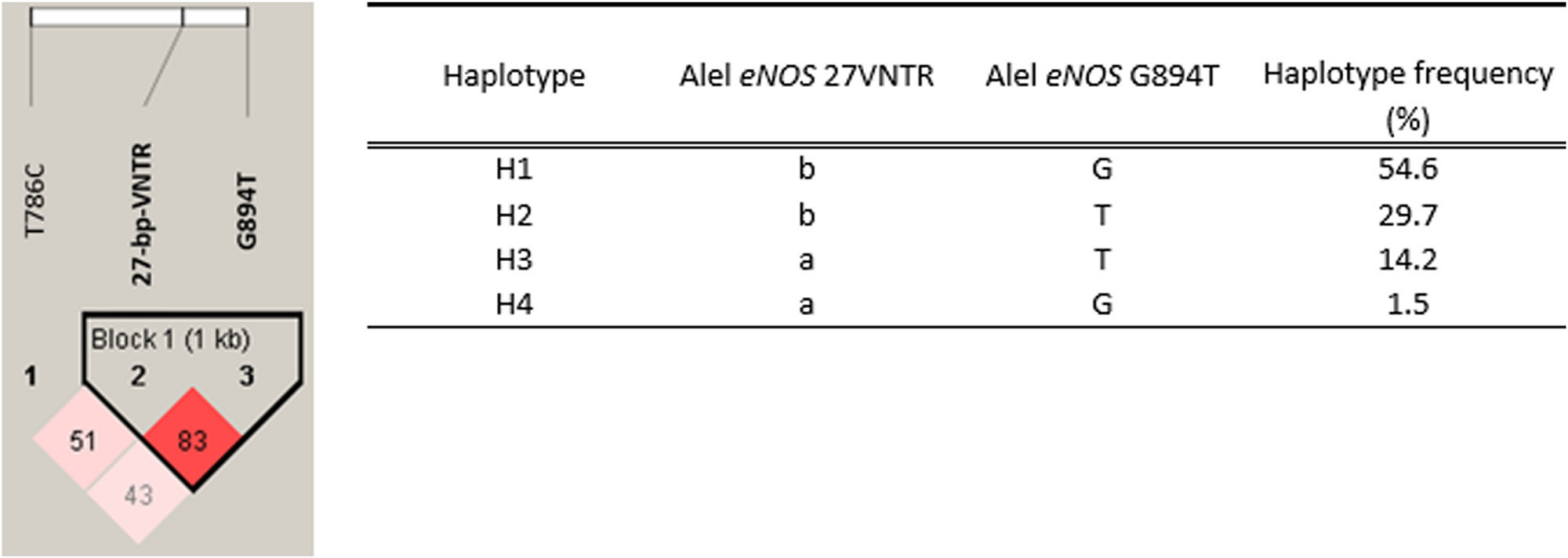
Plot representing linkage disequilibrium of alleles at different positions of eNOS. A linkage disequilibrium plot was generated using the Haploview 4.2 software. Polymorphisms within one haplotype block are indicated in bold. Linkage disequilibrium is displayed for each polymorphism combination as pairwise *D*’ values multiplied by 100. Colour coding represents the magnitude of pairwise LD, with a red-to-white gradient reflecting higher-to-lower LD values.

Analysis using the Haploview program showed that two of the three investigated polymorphisms *eNOS* 27-bp-VNTR and *eNOS* G894T were in linkage disequilibrium (*D*′ = 0.83), whereby four combinations of haplotypes were detected, one of which (H4) had a very low frequency and was therefore excluded from further analyses (Figure 2). There was no statistical difference in the frequency of the combination of haplotypes or carriers of individual haplotypes between unruptured IA patients and patients with aSAH (Table S1). There was also no significant difference in the frequency of combinations of haplotypes or carriers of individual haplotypes between patients with multilobular and those with saccular aneurysms (Table S2).

## 4 Discussion

Analysis of our data revealed a statistically significant association between aSAH and a single eNOS polymorphism (T786C), IA in posterior cerebral circulation, and multilobular IA.

Our sample was comparatively large (almost 300 subjects) and homogenous, as all subjects were Caucasian, European, and non-Finnish (incidence of aSAH being significantly higher in this specific European population, without a higher prevalence of high-risk aneurysm-related factors [5]).

Previous similar studies showed inconsistent results and mostly focused on the presence of IA and less on aSAH [6,7,9,15]. Based on the previous literature, the development of IA and aSAH has a polygenic basis with significant non-genetic risk factor influence [16].

Our results suggest that the CC genotype of the T786C polymorphism is an independent risk factor for aSAH (CC vs T carriers, OR 2.7 (1.189–5.968), CI 95%). The T786C polymorphism has been implicated in the pathogenesis of IA. Paschoal et al. [7] reported a significant association between the T786C polymorphism and the formation of IA, with TT vs TC and CC genotypes showing an increased risk and TT and TC vs CC genotypes showing a protective effect. This suggests that the T786C polymorphism may serve as a predictor for the development of IA in the cerebral vascular system. Contradictorily, while Paschoal et al. [7] established a link between T786C polymorphism and IA formation, it does not address the relationship between this polymorphism and aSAH. Lopes et al. [17] found that the CC genotype was associated with unruptured IAs larger than 12 mm in diameter. This suggests a potential relationship between this genotype and the development of certain aneurysms without directly addressing the risk of rupture [17].

The postulated effects of locally produced (vascular, endothelial) NO are anti-inflammatory and vasodilative. It could consequently be proposed that any lack of these physiological mechanisms due to T786C polymorphism could negatively affect IA growth suppression and IA wall stability, resulting in a higher chance of developing aSAH.

As expected, the data showed a significantly higher rate of rupture in multilobular vs simple saccular IA, as well as in IA in the posterior vs anterior circulation. Studies suggest that irregular-shaped and lobulated IA may be more prone to rupture [18,19]. The anterior cerebral circulation IA may be less likely to rupture if patients receive certain medications, suggesting a complex interplay between aneurysm location, morphology, and systemic factors [20]. There was a significant proportion of ruptured IA with a diameter ≤4 mm (46.5%), which supports observations from our routine practice with ruptured IA but is in disagreement with the widely used risk scores [21,22].

Our study did not reveal any significant differences in the distribution of genotypes and allele carriers for the 27-bp-VNTR and G894T polymorphisms between the groups. This outcome is not surprising, given that previous research on the potential relationship between these polymorphisms and susceptibility to aSAH has often yielded conflicting results, and the associations may vary among different populations [10,23].

The age and aneurysm stratifications in our patients were in accordance with the data reported by Vlak et al. [5]. The results of this meta-analysis demonstrated that the prevalence of aneurysms is higher among individuals aged 50 years or older. It is worth noting that the growth and rupture of aneurysms are more likely to occur after the age of 50.

In the studied sample, women were significantly older than men. The finding of significant age difference in this context might reflect two different previously published findings. The prevalence of AH with aging becomes higher in women than in age-matched men [24]. Given that this data set includes only confirmed IA patients, this could also be in keeping with the reported higher risk of IA formation and aSAH due to relative estrogen deficiency in postmenopausal women.

There was no statistically significant difference between the two groups in our study regarding sex, age, hypertension, and smoking; consequently, none of these factors (or their combinations) were shown to be associated with aSAH.

Each of the aforementioned risk factors has been strongly implicated in aSAH, but these findings have been previously challenged, mostly based on specific demographic groups represented in any given study.

The relationship between sex, hypertension, and smoking with IA rupture is supported by different studies. Zuurbier et al. [25] identified female gender, hypertension, and smoking history as significant risk factors for the rupture of IAs. Furthermore, Kamio et al. [26] and Ya et al. [27] highlighted the role of smoking, particularly nicotine exposure, in increasing the risk of aneurysmal rupture and delayed cerebral ischemia following rupture, respectively. Additionally, Kamio et al. [26] provided a mechanistic insight by demonstrating that nicotine promotes aneurysm rupture through its action on vascular smooth muscle cell α7*-nAChR. Contradicting these findings, Bechstein et al. [28] reported that in the Mongolian population, female gender was not associated with a higher risk of aneurysm rupture, suggesting that the impact of sex on aneurysm rupture may vary with demographic factors. Contradictions arise when considering the role of hypertension in aneurysm rupture. While some studies identify it as a risk factor [29], others do not find it to be an independent risk factor [30]. Additionally, the potential protective role of certain antihypertensive medications, such as β-blockers and statins, is highlighted in the study of Krzyżewski et al. [20], indicating that the relationship between hypertension and aneurysm rupture may be influenced by medication use.

The relationship between smoking and the risk of aneurysm rupture has been the subject of extensive research, with various studies yielding conflicting results. While many studies indicate a strong association between smoking and an increased risk of aneurysm rupture, some research suggests that smoking may not be directly related to the rupture of aneurysms, particularly when considering other confounding factors [31]. One study by Can et al. highlighted the lack of control for certain variables in previous research, such as antihypertensive medication use and family history of aneurysms, which may obscure the true relationship between smoking and aneurysm rupture [32]. This suggests that while smoking is often cited as a risk factor, its role may be confounded by these other factors, indicating that smoking alone may not be the sole contributor to aneurysm rupture. Choi and Park’s research on small ruptured aneurysms indicates that the size and development timeline of aneurysms play a critical role in rupture risk, suggesting that newly developed small aneurysms are less likely to rupture, regardless of smoking status [33]. This finding implies that the relationship between smoking and aneurysm rupture may be more complex than previously thought, as it emphasizes the importance of aneurysm characteristics over lifestyle factors like smoking. Feng et al. conducted a case–control study that supports the notion that smoking may influence aneurysm growth rather than directly causing rupture [34]. Their findings suggest that while smoking is associated with increased aneurysm size, which could theoretically lead to rupture, the direct causative link remains unclear. This aligns with the observations made by Zhang et al., who noted that while tobacco use correlates with certain morphological features of aneurysms, it does not necessarily lead to an increased rupture risk [35].

aSAH patients have a high prevalence of current smoking, but an even higher proportion of these patients have a history of smoking [36]. It is worth noting that the uniformly high prevalence of smoking across groups of our study population could probably be directly related to smoking prevalence in the general population in Croatia (2. European Commission Special Eurobarometer 429: Attitudes of Europeans towards Tobacco and Electronic Cigarettes. 2020. Available online: https://europa.eu/eurobarometer/surveys/detail/2240).

Using the diagnosis of AH broadly as a general risk factor for aSAH could be a possible explanation for the lack of significant correlation in our cohort. Additionally, all study participants were using antihypertensive medications if diagnosed with AH; however, the choice of a specific drug was at the discretion of the clinician involved. Further research using more quantifiable measures of smoking exposure, as well as stratifying study population by the effectiveness of AH treatment in achieving BP goals or specific medications used in treatment, could provide a more detailed insight into the correlation of these factors with aSAH.

The main limitations of our study are both the sample size and the single-centre design. While most of the patients in our country during the specified period were treated in our hospital (high-volume tertiary centre), the results should be interpreted cautiously and validated on a larger sample. The use of this type of data in routine clinical practice, even if taken as a definitive risk factor for aSAH, is hampered by the availability of genetic testing. A definitive diagnosis of unruptured IA leads to a clinical decision about prophylactic occlusion. Risks of complications and mortality rates related to these treatments remain significant factors in decision-making. Current guidelines acknowledge the necessity of further research. Validating the positive result of T786C polymorphism as an independent risk factor for aSAH on a larger scale could allow for a more informed clinical decision-making process for IA treatment.

In summary, our study showed a significantly higher risk of aSAH in patients with IA and T786C eNOS polymorphism, posterior circulation IA, and multilobular IA. Our data analysis did not show a significant correlation between previously reported patient-related risk factors with IA rupture.

## 5 Conclusions

Analysis of our data showed a significant and independent correlation between eNOS genetic polymorphism T786C and aSAH. These results should be verified by larger studies, which would also allow for a more precise estimation of individual risks.

## Supplementary Material

Supplementary Table
