# Supplementary material for: Association of eNOS T786C genetic polymorphism with the risk of aneurysmal subarachnoid haemorrhage
Source: Transl Neurosci. 2025 Apr 16;16(1):20250368. doi: 10.1515/tnsci-2025-0368 (PMC12032989; doi:10.1515/tnsci-2025-0368)
Supplement: Supplementary Table [file tnsci-2025-0368-sm.pdf]

Supplementary material

Table S1: Haplotypes as a risk for SAH. Comparison of the frequency of haplotype combinations and carriers of individual haplotypes with respect to the 27-bp-VNTR and G894T polymorphisms of the eNOS gene in subjects according to the occurrence of aSAH

	aSAH			
	No		Yes	
**Haplotype**	***N***	**%**	***N***	**%**
H1/H1	49	34.3	31	26.7
H1/H2	44	30.8	34	29.3
H2/H2	14	9.8	14	12.1
H2/H3	11	7.7	9	7.8
H3/H1	25	17.5	28	24.1
	$\chi^2 = 2.92$; $p = 0.571$			
Others	25	17.5	23	19.8
H1 carriers	118	82.5	93	80.2
	$\chi^2 = 0.23$; $p = 0.634$			
Others	74	51.7	59	50.9
H2 carriers	69	48.3	57	49.1
	$\chi^2 = 0.02$; $p = 0.887$			
Others	107	74.8	79	68.1
H3 carriers	36	25.2	37	31.9
	$\chi^2 = 1.43$; $p = 0.232$			

N = number of patients, aSAH = aneurysmal subarachnoid haemorrhage.

Table S2: Haplotypes as a risk for multilobular aneurysm formation. Comparison of the frequency of haplotype combinations and carriers of individual haplotypes with respect to the 27-bp-VNTR and G894T polymorphisms of the *eNOS* gene in subjects according to the aneurysm type (saccular vs multilobular aneurysm)

	**Aneurysm type**			
	**Saccular aneurysm**		**Multilobular aneurysm**	
**Haplotype**	***N***	**%**	***N***	**%**
H1/H1	69	31.2	11	28.9
H1/H2	69	31.2	9	23.7
H2/H2	24	10.9	4	10.5
H2/H3	17	7.7	3	7.9
H3/H1	42	19.0	11	28.9
	$\chi^2 = 2.34$; $p = 0.692$			
Others	41	18.6	7	18.4
H1 carriers	180	81.4	31	81.6
	$\chi^2 = 0.00$; $p = 0.985$			
Others	111	50.2	22	57.9
H2 carriers	110	49.8	16	42.1
	χ2 = 0.76; $p = 0.382$			
Others	162	73.3	24	63.2
H3 carriers	59	26.7	14	36.8
	$\chi^2 = 1.65$; $p = 0.241$			

N = number of patients.
